# Genetic structure and distribution of *Parisotoma notabilis* (Collembola) in Europe: Cryptic diversity, split of lineages and colonization patterns

**DOI:** 10.1371/journal.pone.0170909

**Published:** 2017-02-07

**Authors:** Helge von Saltzwedel, Stefan Scheu, Ina Schaefer

**Affiliations:** Georg August University Göttingen, Johann Friedrich Blumenbach Institute of Zoology and Anthropology, Göttingen, Germany; Nanjing Agricultural University, CHINA

## Abstract

Climatic and biome changes of the past million years influenced the population structure and genetic diversity of soil-living arthropods in Europe. However, their effects on the genetic structure of widespread and abundant soil animal species such as the Collembola *Parisotoma notabilis* remain virtually unknown. This generalist and parthenogenetic species is an early colonizer of disturbed habitats and often occurs in human modified environments. To investigate ancient climatic influence and recent distributions on the genetic structure of *P*. *notabilis* we analyzed populations on a pan-European scale using three genetic markers differing in substitution rates. The results showed that *P*. *notabilis* comprises several genetic lineages with distinct distribution ranges that diverged in the Miocene. Genetic distances of COI between lineages ranged between 15% and 18% and molecular clock estimates suggest Late Miocene divergences considering the standard arthropod rate of 2.3% per my. Compared to other soil-living arthropods like oribatid mites, European lineages of *P*. *notabilis* are rather young and genetically uniform. The close association with anthropogenic habitats presumably contributed to rapid spread in Europe.

## Introduction

The ubiquitous soil arthropod species *Parisotoma notabilis* (Schäffer, 1896) is one of the most successful species among Collembola being locally abundant in virtually any habitat in the temperate and boreal zone. Populations can reach densities of up to 10,000 and 6,000 individuals per square meter in forest soils and meadows, respectively, but also typically are present in arable fields, pastures, urban soils and caves [1–8], and even in extreme habitats such as open glacier forelands at high elevation [9]. *P*. *notabilis* is the most abundant Collembola species in Europe [10] and together with *Isotomiella minor* (Schäffer, 1896) it often represents more than 50% of the total individuals in Collembola communities [2,4]. It is morphologically well defined [11–14], but exhibits inter-population differences in tolerance to low pH, mechanical disturbances and metal pollution [1,6]. According to stable isotope ratios of ^15^N/^14^N it feeds as generalist on bacteria, fungi and smaller soil animals including protozoans, nematodes and rotifers [15]. Notably, *P*. *notabilis* reproduces via parthenogenesis, no males have been found in natural populations [16] except for a Swedish population where males rarely occur [17]. Wind dispersal [8], the potential to start populations from a single female individual and generalist feeding make this species a fast and successful colonizer of new and disturbed habitats [18–22].

The genetic structure of *P*. *notabilis* populations is little known except for one study investigating genetic variation within and between European populations [23]. Based on two genetic markers (COI and D2 region of 28S rDNA) they demonstrated that *P*. *notabilis* comprises four different lineages in Europe, with low genetic variance within (<3% for COI and zero for D2) but high variance between lineages (21% for COI and <3% for D2). The authors concluded that these four lineages represent ‘cryptic species’ which evolved independently but without morphological differentiation.

Deep genetic divergences in soil-living arthropods have been described previously and may be due to strong founder effects and genetic bottlenecks after long-distance dispersal combined with limited local dispersal within the soil matrix [24]. Ancient divergences and survival in small patches during the Quaternary Ice-Ages may also generate patterns of deep divergence in *P*. *notabilis*, however, these questions have not been addressed yet.

In order to investigate the relevance of founder effects and historical dispersal patterns, we extended the geographic sampling of the previous study [23] and included western Russia, the Ukraine, Turkey, the Balkan Peninsula, Norway, Great Britain and Greenland. Thus, the sampling included areas of northern Europe that were covered by glaciers during the last Ice Age, i.e. regions that must have been colonized in the Holocene by *P*. *notabilis*, resulting in populations of low genetic variation. Colonization likely occurred from southern Europe and south-eastern Russia, similar to the grasshopper *Chorthippus parallelus*, the hedgehog *Erinacues europeaus*, the bear *Ursus arctos*, the alder *Alnus glutinosa* and oaks *Quercus* spp. [25–27]. Therefore, we expected northern lineages to be closely related to lineages of western and central Europe, but distantly to those south of the Alps.

We used three genetic markers, the mitochondrial COI gene, the D3-D5 region of 28S rDNA and the nuclear gene Histone H3 that provided resolution intermediate to 28S and COI. This is the first study comparing two protein-coding genes and one ribosomal gene with different mutation rates to detect recent and old diversifications, independent evolutionary units (IEUs) and colonization patterns of Collembola in Europe.

## Materials and methods

### Ethics statement

Sampling sites were outside Nature Reserve Areas and no permission for soil samples was required. The field study did not involve any endangered or protected species.

### Sampling of animals and DNA extraction

**Fig 1:**
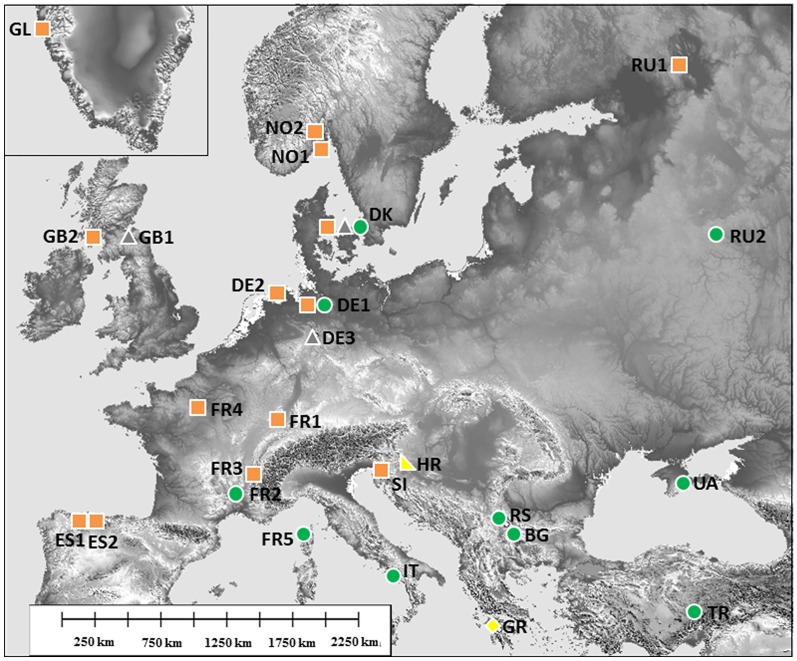
Sampling locations and distribution of lineages of *Parisotoma notabilis*. Genetic lineages were named following Porco et al. (2012), lineage L0 (grey triangles) occurs in three sampling locations (DE1, DK, GB1), while lineages L1 (green circles) and L2 are widespread in the southwest and the east of Europe, respectively. Lineages L3 (yellow diamond) and L4 (yellow turned triangle) are geographically isolated in the south of Europe.

Leaf litter and humus layers from about two square meters of deciduous and coniferous forests was collected in 26 locations in Europe, including Greenland, northwest Russia (Karelia), Ukraine, Turkey, the Balkan region (Bulgaria, Serbia, Croatia, Greece), Italy, Spain, and transferred to the University of Göttingen (Fig 1, Table 1).

**Table 1:** Countries and sampling locations of *Parisotoma notabilis*.

country	location	abbreviation	n inds	IEUs (lineages)
**Denmark**	Humlebaek	DK	5	0, 1, 2
**Great Britain**	Melrose	GB1	5	0
	Island of Arran	GB2	5	2
**Greenland**	Kobbefjord / Nuuk	GL	5	2
**Norway**	Rod	NO1	4	2
	Skjervenmoen, Fössa Öst	NO2	4	2
**Russia**	Petrozavodsk, Karelia	RU1	5	2
	Znamenskoe	RU2	5	1
**France**	Voegtlinshoffen	FR1	4	2
	Salavas	FR2	5	1
	Chartreuse	FR3	5	2
	Rambouillet	FR4	4	2
**Germany**	Uelzen—Elbeseitenkanal	DE1	5	1, 2
	Norden	DE2	4	2
	Solling, Neuhaus	DE3	5	0
**Bulgaria**	Bosnek	BG	5	1
**Croatia**	Sljeme	HR	5	4
**France**	Korsika, Olmi-Capella	FR5	5	1
**Greece**	Chrysovitsi	GR	5	3
**Italy**	Felitto	IT	4	1
**Serbia**	Sreckovac	RS	5	1
**Slovenia**	Postojna	SI	3	2
**Spain**	Oviedo	ES1	3	2
	Ponga	ES2	5	2
**Turkey**	Kayseri	TR	5	1
**Ukraine**	Kubalach, Crimea	UA	5	1
	**total no. of individuals.**		**120**	

Individuals were collected in south, central and northern Europe. The assigned genetic lineages (IEUs) are listed for all analyzed individuals of all sampling locations.

Animals were extracted by heat, collected in water [28], transferred into 96% EtOH and stored at -20°C until further analyses. For species identification specimens were sorted under a dissecting microscope and determined by light microscopy following [29]. Genomic DNA was extracted from single individuals of *P*. *notabilis* (n = 120) using the DNeasy^®^ Blood and Tissue Kit (Qiagen, Hilden, Germany) following the manufacturer’s protocol for animal tissue. Purified DNA was eluted in 30 μl buffer AE and stored at -20°C until further preparation. Two nuclear genes, Histone H3 and the D3-D5 region of 28S rDNA, and the barcoding fragment of the mitochondrial COI gene were amplified in 25 μl volumes containing 12.5 μl SuperHot Taq Mastermix (Genaxxon Bioscience GmbH, Ulm, Germany) with 1.5 μl of each primer (10 pM), 4.5 μl H_2_O, 2 μl MgCl_2_ (25 mM) and 3 μl template DNA. A 374 bp fragment of the protein coding gene H3 was amplified, using the primers H3F1 5’-ATG GCT CGT ACC AAG CAG ACV GC-3’ and H3R1 5’-ATA TCC TTR GGC ATR ATR GTG AC-3’ [30]. A ~573 bp fragment of the nuclear 28S rDNA was amplified using the primers 28Sa 5’-GAC CCG TCT TGA AGC ACG-3’ and 28Sbout 5’-CCC ACA GCG CCA GTT CTG CTT ACC-3’ [31]. For the 709 bp fragment of the COI gene the primers LCO1490 5’-GGT CAA CAA ATC ATA AAG ATA TTG G-3’ and HCO2198 5’-TAA ACT TCA GGG TGA CCA AAA AAT CA-3’ [32] were used. PCR conditions included one initial activation step at 95°C for 15 min, followed by 35 amplification cycles of denaturation at 94°C for 15 s, annealing at 45°C (COI) or 49°C (28S) or 59°C (H3) for 15 s, elongation at 72°C for 15 s and a final elongation step at 72°C for 6 min. Positive PCR products were purified with the QIAquick PCR Purification Kit (Qiagen, Hilden, Germany) following the manufacturer’s protocol, eluted in 30 μl HPLC water and sent for direct sequencing to the Göttingen Genome Laboratory (Institute for Microbiology and Genetics, Georg August University of Göttingen). All sequences are available at GenBank (S1 Table).

Genomic DNA was extracted from entire specimens but secondary vouchers (same morphological species from the same population) were deposited at our collections at J.F. Blumenbach Institute of Zoology and Anthropology, Georg August University Göttingen, Germany.

### Data analyses

Sequences were edited, ambiguous positions were corrected by hand and nucleotide sequences were translated into amino acid sequences using the invertebrate mitochondrial code (COI) and the standard code (H3) implemented in Sequencher v4.10 (Gene Codes Corporation, USA). Nucleotide (28S) and protein sequences (COI and H3) were aligned separately and as combined matrix (concatenated sequences of all three genes) with Clustal W [33] implemented in BioEdit v7.0.1 [34]; protein alignments were retranslated to nucleotide sequences.

The best fit model of sequence evolution for each alignment (COI, 28S, H3, combined matrix) was inferred according to the hLRT in TOPALi v2.5 [35] using the PHYML algorithm. Phylogenetic trees were calculated with Maximum Likelihood in RAxML v8.0.0 [36] and Bayesian Inference (BI) in MrBayes v3.1.2 [37]. Phylogenetic analyses were performed for single genes (28S, COI, H3) and the combined matrix. The model of sequence evolution was GTR+I+Γ for the COI and the combined matrix, GTR+ Γ for 28S and JC for the H3 matrix. For ML analyses, parameters were GTRGAMMAI and 10,000 bootstrap replicates. For Bayesian inference lset parameters were nst = 6, rates = invgamma for the combined and the COI matrix, nst = 6, rates = gamma for 28S and nst = 1 for H3. Two independent MCMC chains were run for ten million generations that were sampled every 1,000^th^ generation, a burnin of 2,500 was applied. To consider the different substitution rates of single codon positions, i.e. the first and second nucleotide in a codon being more conserved than the third, we also ran one analysis in MrBayes with the combined matrix that used the implemented codon model M3 for the two protein coding genes in the combined matrix. Running the M3 model for two codon partitions increased the calculation times considerably, and we additionally used a quicker approach that still considered variable substitution rates for codon positions. Here the third codon position of the COI partition was excluded from the analysis and the M3 model only applied to the H3 partition. In mitochondrial genes the third codon position is assumed to be four-fold degenerated [38] and therefore effectively neutral. The exact settings for all MrBayes runs are provided in the legend of S4 Fig.

For all COI sequences obtained in this study (120 individuals, 709 bp), the number of independent evolutionary units (IEUs) was inferred with a GMYC (general mixed Yule-coalescent) analysis [39–41]. However, in parthenogenetic genomes without recombination all loci are linked and random genetic drift and selection act differently on parthenogenetic organisms. To test for parthenogenetic speciation, we also applied the 4x rule which has been empirically tested on asexual organisms [42,43]. We used the K/θ < 4 threshold between clades to delimitate parthenogenetic species, i.e. the ratio of between and within well supported clades. The estimator K was calculated as observed sequence distance between clades corrected for multiple hits (GTR+I+G estimated with TOPALi). The estimator θ was calculated as π/(1–4π/3), with π being the within clade nucleotide diversity (S2A Table). Calculations were done in an Excel spreadsheat.

For GMYC analysis, an ultrametric tree was generated in BEAST v1.8.0 [44] with GTR+I+Γ as model of sequence evolution. The MCMC chain was run for 500 million generations and sampled every 5,000^th^ generation and a burnin of 2,500 was applied. The GMYC analysis was performed with the splits package 1.0–19 [44] in R v3.1.0 (R Development Core Team, 2008) using single threshold delimitation [40, 41].

Molecular divergence times of major lineages were estimated with a molecular clock analysis in BEAST v1.8.0 [45] based on three datasets to account for differences in tree topologies and substitution rates of nuclear genes and to account for the greater genetic variance when combining our data with the COI dataset of [23]: First, for the combined matrix of three genes, we set a fixed substitution rate of 0.0115 for the COI partition and relaxed substitution rates of the H3 and 28S rDNA partitions that were estimated by BEAST. Second, for the COI alignment of 120 individuals from this study with a length of 709 bp, we set a strict substitution rate of 0.0015 that corresponds to the common invertebrate rate of COI of 0.023 substitutions per site per million years [38,46]. Third, for the combined COI alignment that included 123 additional individuals from [23] with had a length of 500 bp, all parameter settings were the same as for analysis (2). We used the Yule process as tree prior [47] for all analyses because it allows higher rate variance among branches, which appeared to be more appropriate for this parthenogenetic species. Preliminary analyses showed faster convergence and better likelihoods for the Yule process compared to analyses using coalescent tree priors. Convergence of the MCMC chain after 600 million generations (sampled every 60,000^th^ generation) with a burnin of 2,500 was confirmed using Tracer v1.4 [48].

The number of nucleotide and protein haplotypes was determined using the online tool FaBox v1.41 [49]. Haplotype alignments of the amino acid sequences for COI and H3 and the nucleotide sequences of 28S were generated with FaBox v1.41, each gene was checked by eye for lineage specific substitutions. Haplotype based alignments were used for GMYC and MrBayes analyses. Lineage assignments corresponded to the IEU estimated by the GMYC analysis. Analysis of molecular variance (AMOVA) and genetic distance analyses (uncorrected p-distances) were performed separately for sampling locations and lineages and each gene in ARLEQUIN v3.5 [50] with 20,000 permutations.

## Results

### Phylogeny and independent evolutionary units

**Fig 2:**
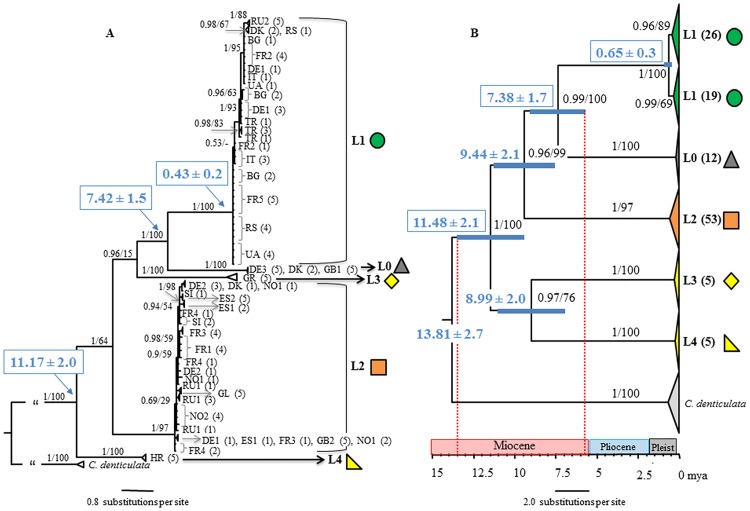
Phylogenetic relationships and molecular divergence estimates of five European lineages of *Parisotoma notabilis*. A Bayesian phylogeny based on nucleotide sequence data of three genes (28Sr DNA, H3 and COI), blue values in boxes are divergence estimates based on the combined matrix calculated with BEAST (see also S3 Fig). **B** Molecular clock tree based on COI sequences (709 bp) calculated with BEAST. Blue bars on nodes represent 95% confidence intervals. Bold numbers on nodes are divergence times with confidence intervals in mya. A geological timescale is provided below the tree, indicating the Miocene (23–5.3 mya), the Pliocene (5.3–1.8 mya) and the Pleistocene (Pleist. 1.8–0.0114 mya). Dashed red lines indicate radiations of major lineages during the Miocene. Terminal clades are collapsed; numbers in brackets indicate the numbers of individuals of each sampling locations included in the respective clades. Numbers on nodes are posterior probabilities (Bayesian Inference) and bootstrap values (Maximum Likelihood).

The phylogenetic tree based on the combined matrix of the three genes gave the best resolution and statistical support for internal and terminal nodes (Fig 2A); topologies of ML and BI trees were very similar. The phylogenetic trees based on the combined matrix and the individual genes were always congruent i.e., COI haplotypes were linked with specific H3 and 28S alleles.

The GMYC analysis estimated five IEU for the COI tree (Fig 2B) with a threshold of <1% genetic distances as cut off. The number of ML entities was 7 with entities number 6 and 7 being outgroups. The likelihood ratio between the null model and the GMYC model was 21.05 (p<0.001). Branching patterns in the phylogenetic analyses with the single genes H3 and 28S (S1 Fig) and the combined dataset (Fig 2A) were consistent with these IEUs and corresponded to highly supported clades (posterior probabilities = 1, bootstrap = 97–100) in all four datasets. Results of the 4x rule, however, indicated that all IEU belong to a single parthenogenetic species (S3 Table).

All populations clustered with L0-3 from [23] and were named accordingly, except for the population from Greece which was not identical, but formed a sister taxon to L3 from Paris, and the population from Croatia (L4) which differed considerably from all other lineages. Notably, the COI sequences of two individuals of L1 [23] clustered within L2 (S2 Fig**)**.

L0 comprised only three sampling locations in central and northern Europe, whereas L1 and L2 comprised large phylogeographic clades, covering wide regions in south and central Europe to the east (L1) and in western Europe from south to north (L2). Different lineages coexisted only in two sampling locations, in Denmark and Germany (Uelzen); however, each of the three German sampling locations was occupied by different lineages. The four French sampling locations comprised only two different lineages, with L1 being present only in southern France (FR2 and FR5) and L2 being present in all other French sampling locations. Further, two different lineages were sampled in Scotland; L0 was present in the east of Scotland (GB1) while the widespread L2 was present in the west of Scotland (GB2) (Figs 1 and 2). Topologies of the Bayesian trees calculated with the combined matrix and the codon models (S4 Fig) differed only slightly in the order of some terminal branches but relationships among lineages were identical with the tree calculated with the GTR model (Fig 2A). Posterior probabilities of the backbone, i.e. relationships among lineages, changed in the different trees, but not the node support of lineages which was always 1.

### Genetic diversity

Genetic distances between the lineages 0 to 5 (Table 2) were very high, ranging between 15% and 18% for the COI gene, between 5% and 11% for H3 and 0.5% to 1.9% for 28S. Genetic distances between sampling locations were also high, ranging for COI between 9% and 17%, for H3 between 4% and 7% and for 28S between 0.7% and 1% (Table 3).

**Table 2:** Mean genetic distances (observed p-distances in %) of *Parisotoma notabilis* in Europe for three genes.

	COI					H3					28S				
Lineage	L0	L1	L2	L3	L4	L0	L1	L2	L3	L4	L0	L1	L2	L3	L4
**Lineage 0**	0					0					0				
**Lineage 1**	15.8	0.66				4.55	0.17				1.22	0			
**Lineage 2**	16.64	15.94	0.48			6.42	6.68	0.05			1.22	1.39	0		
**Lineage 3**	18.34	17.49	17.35	0.34		6.95	6.15	6.95	0.96		0.7	0.87	0.52	0	
**Lineage 4**	17.21	17.91	15.23	16.08	0.28	7.22	6.95	8.02	10.7	0	1.74	1.92	1.22	1.05	0

Genetic distances between genetic lineages (L0-L4) of *Parisotoma notablilis*. Genetic lineages refer to independent evolutionary units (IEUs) identified with GMYC.

**Table 3:** Mean genetic distances (observed p-distances in %) of *Parisotoma notabilis* between and within locations in Europe for three genes.

B		between locations			within locations		
location		COI	H3	28S	COI	H3	28S
DK	**Denmark**	12.21 ± 2	4.38 ± 1	0.94 ± 0	13.17	4.28	1.01
GB1	**Great Britain**	15.52 ± 4	5.15 ± 2	1.15 ± 0	0	0	0
GB2		8.95 ± 8	3.94 ± 3	0.70 ± 1	0	0	0
GL	**Greenland**	8.96 ± 8	4.19 ± 3	0.70 ± 1	0.16	0	0
NO1	**Norway**	9.01 ± 8	3.94 ± 3	0.70 ± 1	0.73	0	0
NO2		8.82 ± 8	3.94 ± 3	0.70 ± 1	0.14	0	0
RU1	**Russia**	8.91 ± 8	5.15 ± 2	0.70 ± 1	0.2	0	0
RU2		11.43 ± 7	4.42 ± 3	0.92 ± 1	0	0.16	0
FR1	**France**	9.01 ± 8	3.94 ± 3	0.70 ± 1	0.07	0	0
FR2		11.45 ± 7	4.30 ± 3	0.92 ± 1	0.45	0	0
FR3		8.85 ± 8	3.94 ± 3	0.70 ± 1	0.34	0	0
FR4		9.02 ± 8	3.94 ± 3	0.70 ± 1	0	0	0
DE1	**Germany**	10.53 ± 5	4.09 ± 2	0.86 ± 0	6.54	2.67	0.56
DE2		9.14 ± 8	3.94 ± 3	0.70 ± 1	0.35	0	0
DE3		15.52 ± 4	5.15 ± 2	1.15 ± 0	0	0	0
BG	**Bulgaria**	11.00 ± 7	4.23 ± 3	0.92 ± 1	0.73	0	0
HR	**Croatia**	12.31 ± 2	4.42 ± 1	0.94 ± 0	0.28	0	0
FR5	**France**	10.74 ± 7	4.23 ± 3	0.92 ± 1	0	0	0
GR	**Greece**	17.41 ± 0	6.74 ± 1	0.69 ± 0	0.34	0.96	0
IT	**Italy**	10.93 ± 7	4.23 ± 3	0.92 ± 1	0.56	0	0
RS	**Serbia**	10.86 ± 7	4.22 ± 3	0.92 ± 1	0.51	0.11	0
SI	**Slovenia**	8.99 ± 8	3.94 ± 3	0.70 ± 1	0.09	0	0
ES1	**Spain**	9.19 ± 8	3.94 ± 3	0.70 ± 1	0.66	0	0
ES2		9.08 ± 8	3.94 ± 3	0.70 ± 1	0	0	0
TR	**Turkey**	11.09 ± 7	4.23 ± 3	0.92 ± 1	0.2	0	0
UA	**Ukraine**	10.86 ± 7	4.23 ± 3	0.92 ± 1	0.51	0	0

Within sampling locations genetic distances were generally low or non-existing, except for the population from Denmark with 13% (4% in H3 and 1% in 28S) and Germany (DE1) with 7% distance in COI (3% in H3 and 0.6% in 28S), the only locations with coexisting lineages. Accordingly, molecular variance was very high between locations with 90% variance for COI, 92% for H3 and 92% for 28S and low within locations with 10% variance for COI, 8% for H3 and 8% for 28S (Table 4).

**Table 4:** Analysis of molecular variance (AMOVA) among and within sampling locations of the *Parisotoma notabilis* sampled across Europe, based on sequence variance of three genes.

	COI		H3		28S	
source of variation	between locations	within locations	between locations	within locations	between locations	within locations
df	25	94	25	94	25	94
sum of squares	4227.64	355.88	917.64	61.2	269.93	18
variance components	35.85 Va***	3.79 Vb***	7.82 Va***	0.65Vb***	2.30 Va***	0.19 Vb***
% variation	90.45	9.55	9.31	7.69	92.31	7.69
fixiation indices	Fst 0.904***		Fst 0.923***		Fst 0.923***	

Asterisks indicate significant differences at p,0.05; df, degrees of freedom; Fst, F-statistics.

Haplotype diversity of the mitochondrial gene was moderately high. The 120 sequenced individuals of *P*. *notabilis* separated for COI into 39 nucleotide and 18 amino acid haplotypes, the nuclear genes had twelve nucleotide and two amino acid haplotypes of H3 and five nucleotide haplotypes of 28S. The amino acid haplotypes of COI had at least one non-synonymous and lineage specific substitution in each lineage (S4 Table). L1 and L2 were more variable, separating into sublineages, i.e. into regional lineages with different amino acids only being present in Bulgaria, Germany (Uelzen), south and central France (FR1-2, FR4), near Moscow (RU2) and Turkey. The H3 gene had two non-synonymous and lineage specific substitutions between L4 and all other lineages and several lineage specific nucleotide substitutions. Several nucleotide substitutions in 28S were lineage specific and affected all individuals of a lineage (S3 Table).

### Molecular divergence times

Estimates of divergence times refer to the COI dataset including all sequences of this study (Fig 2B) because this alignment contained more informative sites compared to the combined alignment with sequences of [23]. According to a constant substitution rate of 2.3% per million years [38,47], the five lineages diverged in the Miocene, 11.5–7.4 mya. The widespread south European L1 and the locally occurring L0 from central Europe were the youngest, with a Late Miocene origin about 7.4 ± 2.0 mya. The western European L2 and the southern European L3 and L4 diverged about 9.4 and 9.0 ± 2.0 mya, respectively, according to the phylogeny of COI. Despite the ancient diversifications of lineages, populations at sampling locations were rather young, between 0.1 and 0.023 my old, and of Pleistocene origin. Divergence time estimates of the combined matrix (S3 Fig) were very similar to the COI based divergences (Fig 2) although tree topology differed and was very similar to the Bayesian trees (Fig 2A, S4 Fig). The tree topology of the BEAST analysis and the combined matrix only differed in the relationship of L4 that was sister lineage to L2 and not to all other lineages.

Molecular clock analyses based on the COI dataset of the present study and of [23] differed from the molecular clock estimates above in having lower node supports of the phylogenetic groups, a lower resolution of the phylogenetic backbone and lineages separated much earlier (S2 Fig). However, radiations of lineages also occurred in the Miocene (17.8–13.0 mya), but were considerably older than in the analyses with fewer taxa and longer sequences (11.5–7.4 mya).

## Discussion

This study analyzed the phylogeographic structure of *P*. *notabilis* in Europe, one of the most widespread and abundant species of Collembola. The sampling region covered southern, central and northern Europe from east (Ukraine) to west (Pyrenees). Four of the five genetic lineages of this study corresponded with the lineages of [23] but had a wider distribution range due to the extended sampling area. Combined with our data, the southern European lineage L1 of [23] is also common in the east of Europe (Russia2, Ukraine and Turkey), and lineage L2, assumed to be restricted to the Alpine-Carpathian mountain ranges by [23], in fact is widely distributed in western and northern Europe (France, Denmark, Norway, Greenland, west of Scotland) and the Pyrenees. Interestingly, these two widespread lineages are parapatric, indicating either the importance of northern Spain as refuge area during the Last Ice Age or the existence of a contact zone of two lineages [26,51–57] with otherwise distinct distribution ranges. Lineage L0 likely is the type-species lineage and only occurred in three of our sampling locations. However, in combination with the data of [23], it appears to have a rather continuous distribution range in the vicinity of The Channel, and along the coasts of North- and Baltic Sea. Further, additional to the previously described lineage L3 that only occurred in Paris (France) [23], we identified a lineage distantly related to this population in southern Europe (Greece) and a new lineage from a single location in Croatia. These lineages add to the cryptic genetic diversity of *P*. *notabilis* in Europe, in particular in southern Europe.

Each of the five lineages of *P*. *notabilis* identified in this study had specific, non-synonymous substitutions in the COI gene and coexistence of lineages was rare, indicating selection and subsequent spread of the most competitive genotype. However, all lineage specific substitutions in the H3 gene were synonymous, suggesting that fixation of alleles due to bottlenecks and founder events also contributed to the high genetic structure between populations. Interestingly, lineages L0-L2 co-occurred in a small region in Canada [23], either due to multiple, independent anthropogenic introductions and/or due to different ecological conditions, allowing coexistence of lineages. This suggests that in Canada, compared to Europe, populations are more dynamic either because competitive exclusion among haplotypes is in progress due to recent establishment of *P*. *notabilis* or because strong (abiotic) disturbances structure populations facilitating maintenance of genetic variance within sampling locations.

The molecular divergence estimates indicated three radiation events. First, in the Late Miocene, the separation of the widespread lineages L0-L2 (11.5 mya) and the divergences into the five IEUs (L0-L4; 9–7.4 mya) occurred. Second, much later in the Pleistocene, the separation of lineage L1 into two sublineages occurred (0.65 mya). These radiation events coincide with climatic and biotic changes in Europe, i.e. changes from warm and wet climate during the Miocene and the extension of grassland together with the establishment of deciduous forests in Europe in the Late Miocene and Pliocene [58–65]. This suggests that *P*. *notabilis* benefitted from colder climatic conditions in the Late Miocene and associated changes in vegetation from grassland to woodland which allowed to expand its range size considerably.

Despite the wide ranges of lineages, coexistence was very restricted; only five locations in Europe were colonized by two or more lineages of *P*. *notabilis*; two sites in France (Paris and Le Port, Ariege), one in Spain (Gerona, Catalonia) [23], one in Germany (Uelzen) and one in Denmark (Humblebaek). These sampling sites were close to urban areas suggesting that anthropogenic transport and disturbance favor coexistence of lineages. Generally, *P*. *notabilis* occurs in anthropogenic and disturbed habitats, suggesting a synanthropic distribution, i.e. passive dispersal by humans and establishment in human associated agricultural or managed systems.

Overall, results of the present study show that the ubiquitous Collembola species *P*. *notabilis* comprises several genetic lineages with distinct distribution ranges of Miocene and Pliocene origin. If genetic lineages represent cryptic species with genetic but no morphological divergence remains to be tested. However, as indicated by the 4x rule the intraspecific variance of parthenogenetic exceeds that in sexual species. Other studies also indicate that intraspecific genetic variance of COI is high in Collembola species [66] and other species of soil invertebrates, with intra-specific sequence divergences ranging between 11% and 32% [23, 24, 67–69]. More investigations on life-history traits, fitness differences and ecological preferences of distinct lineages are required to clarify this issue. Compared to other species of Collembola [70], European lineages of *P*. *notabilis* are rather young, genetically uniform and depauperate. The human association of this species likely enabled rapid spread of few or single individuals, likely resulting in founder effects and the establishment of genetically homogenous lineages. Thereby, *P*. *notabilis* is an interesting model organism to investigate population dynamics, adaptations and fitness differences among IEUs. Further, its cosmopolitan distribution enables to compare these processes in independent geographic regions including Europe and North America, as the same lineages occur on both continents but gene flow is impeded between the continents. In contrast to many soil-living organisms, *P*. *notabilis* is easy to culture and has short generation times, making this species an ideal model organism for studying evolutionary processes and population genetics of soil invertebrates in both the field and laboratory.

## Supporting information

S1 Fig Bayesian phylogeny of European lineages of *Parisotoma notabilis* based on single genes. (A) Nucleotide sequences of the H3 gene and (B) the 28S rDNA (D3-D5 region). Numbers on nodes are posterior probabilities and bootstrap values.
(TIF)

S2 Fig Bayesian trees based on COI sequences of *Parisotoma notablilis* from Europe. Molecular divergence estimates of European of *P*. *notabilis* calculated with BEAST based on a 500 bp alignment including all COI sequences from this study (n = 120) and from Porco *et al*. (2012) (n = 123). Numbers of different haplotypes (*HT*) are indicated in brackets next to the genetic lineage, * indicate lineages and haplotypes from Porco *et al*. (2012); dashed lines indicate radiations of major lineages in the Miocene (red) and Miocene-Pliocene (blue). Note that divergence estimates are several million years older than in 2B.
(TIF)

S3 Fig Molecular divergence estimates of European *Parisotoma notabilis* calculated with BEAST based on the combined alignment. The combined alignment included 28S rDNA, COI and H3, for age estimation we useda strict clock with a substitution rate of 2.3% for COI and estimated substitution rates for the other genes. The topology differs slightly from the COI based phylogenetic trees but divergence estimates are very similar to those calculated with the 709 bp COI fragment presented in. 2B.
(TIF)

S4 Fig Relationships among genetic lineages of *Parisotoma notabilis* in Europe based on Bayesian inference and the combined matrix and using codon models for the protein coding partitions. **(**A) The M3 model applied to COI and H3, the mitochondrial code was set for the COI partition and the universal code for H3 (mrbayes block settings: outgroup 43; charset D3 = 1–577; charset COI = 578–1285; charset H3 = 1286-.; partition by_gene = 3: D3, COI, H3; set partition = by_gene; lset applyto = (1) nucmodel = 4by4 code = universal nst = 6 rates = gamma; lset applyto = (2) code = metmt rates = invgamma; lset applyto = (3) code = universal nst = 1; lset applyto = (2,3) nucmodel = codon omegavar = M3; mcmc ngen = 1000000 samplefreq = 100; end; (B) The codon model M3 applied only for the nuclear coding gene (H3), for the mitochondrial COI gene the third codon position was excluded; this speeded up calculation times considerably, compared to the model of A (mrbayes block settings: exclude 578–1285\3; lset applyto = (1, 3) code = universal; lset applyto = (1) rates = gamma; lset applyto = (1,2) nucmodel = 4by4 nst = 6; lset applyto = (2) code = metmt rates = invgamma; lset applyto = (3) nst = 1 nucmodel = codon omegavar = M3; mcmc ngen = 1000000 samplefreq = 100; end;). Settings of the Bayesian tree in Fig 2A were: lset applyto = (1) nst = 6 rates = gamma; lset applyto = (2) nst = 6 rates = invgamma; lset applyto = (3) nst = 1; mcmc ngen = 10000000 samplefreq = 1000; end;
(TIF)

S1 Table Accessionnumbres of DNA sequences of *Parisotoma notabilis* from Europe obtained in this study. All sequences are available at NCBI GenBan. Countries, sampling locations and sampling coordinates are listed.
(PDF)

S2 Table Values to estimate speciation among parthenogenetic lineages (Birky's 4x rule) for the five genetic lineages of *Parisotoma notabilis* sampled in Europe. Lineages correspond to nodes connecting clades in Fig 2B, Lineages 1.1 and 1.2 are the two sister clades of Lineage 1 with 26 (L1.1) and 19 (L1.2) individuals. Nucleotide diversity (π), pairwise difference between sequences (d), number of individuals (n), sequence length (L) and nucleotide diversity estimator (θ). Calculations are based on genetic distances and the phylogeny generated with 120 individuals and a 709 bp fragment of COI. The estimator of nucleotide diversity θ was calculated as π/(1–4π/3).
(PDF)

S3 Table K/θ between highly supported clades of *Parisotoma notabilis* from Europe to estimate K for Birky's 4x rule. K/θ ≥ 4 indicate that samples are from different species and K/θ ≤ 4 indicate that samples are from the same species. K is the observed sequence distance d from S2 Table corrected for multiple hits using GTR+G+I as estimated by TOPALI.θ values correspond to S2 Table, if θ of two clades differed, the larger value was used.
(PDF)

S4 Table Positions of lineage specific substitutions in the alignments of three genes (COI, Histone 3, 28S rDNA) of 120 individuals of *Parisotoma notabilis* sampled across Europe. For COI, the amino acid alignment (aa) was analyzed, for Histone 3 the nucleotide (nct) alignment was investigated as all amino acid sequences were identical. The common characters (amino acid for COI, nucleotide for Histone 3 and 28S rDNA) are listed next to the specific substitution.
(PDF)
