# Supplementary figures and images for: Genetic structure and distribution of Parisotoma notabilis (Collembola) in Europe: Cryptic diversity, split of lineages and colonization patterns
Source: PLoS One. 2017 Feb 7;12(2):e0170909. doi: 10.1371/journal.pone.0170909 (PMC5295681; doi:10.1371/journal.pone.0170909)

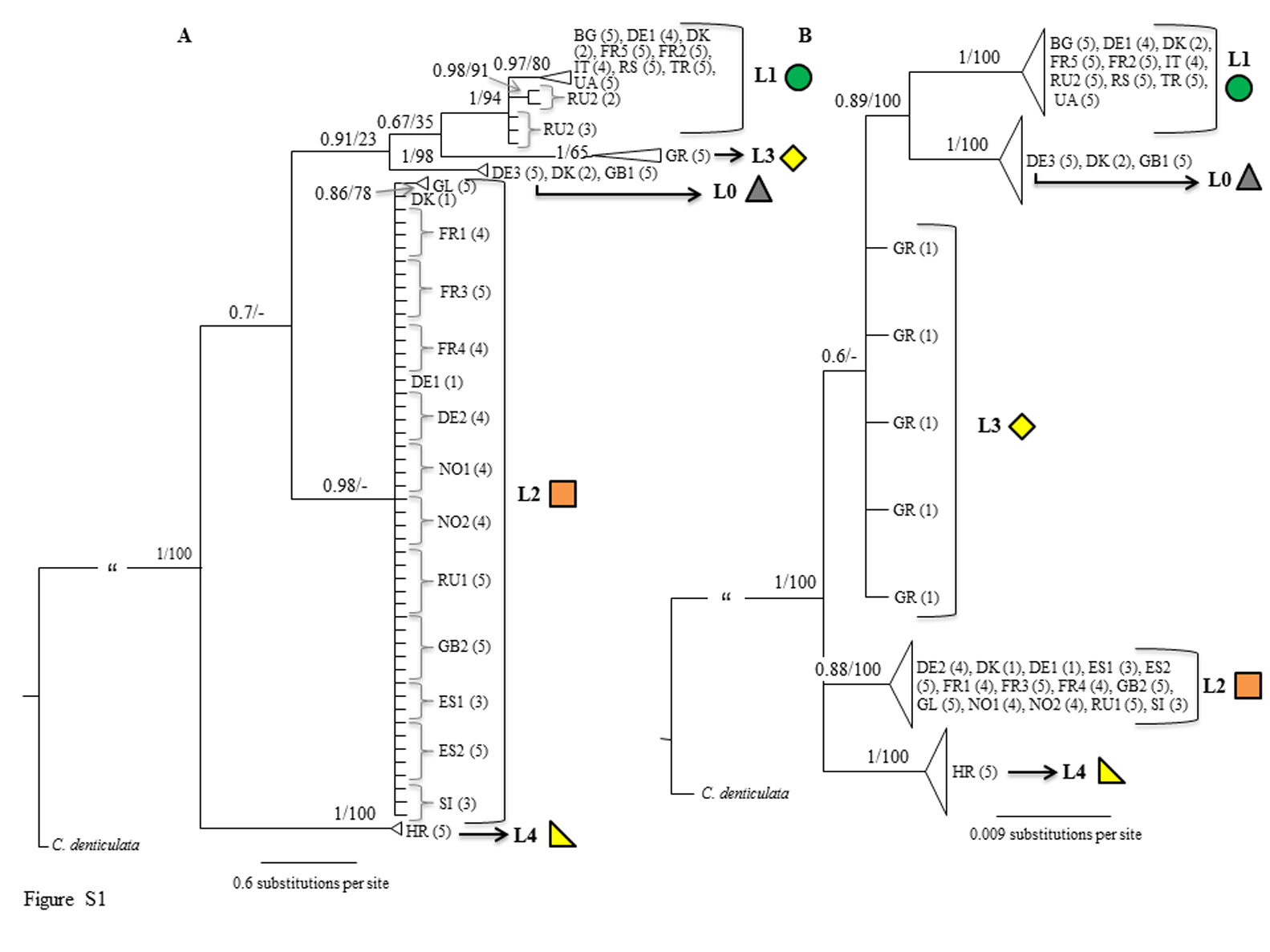

Supplement: S1 Fig — (A) Nucleotide sequences of the H3 gene and (B) the 28S rDNA (D3-D5 region). Numbers on nodes are posterior probabilities and bootstrap values. (TIF) [file pone.0170909.s001.tif]

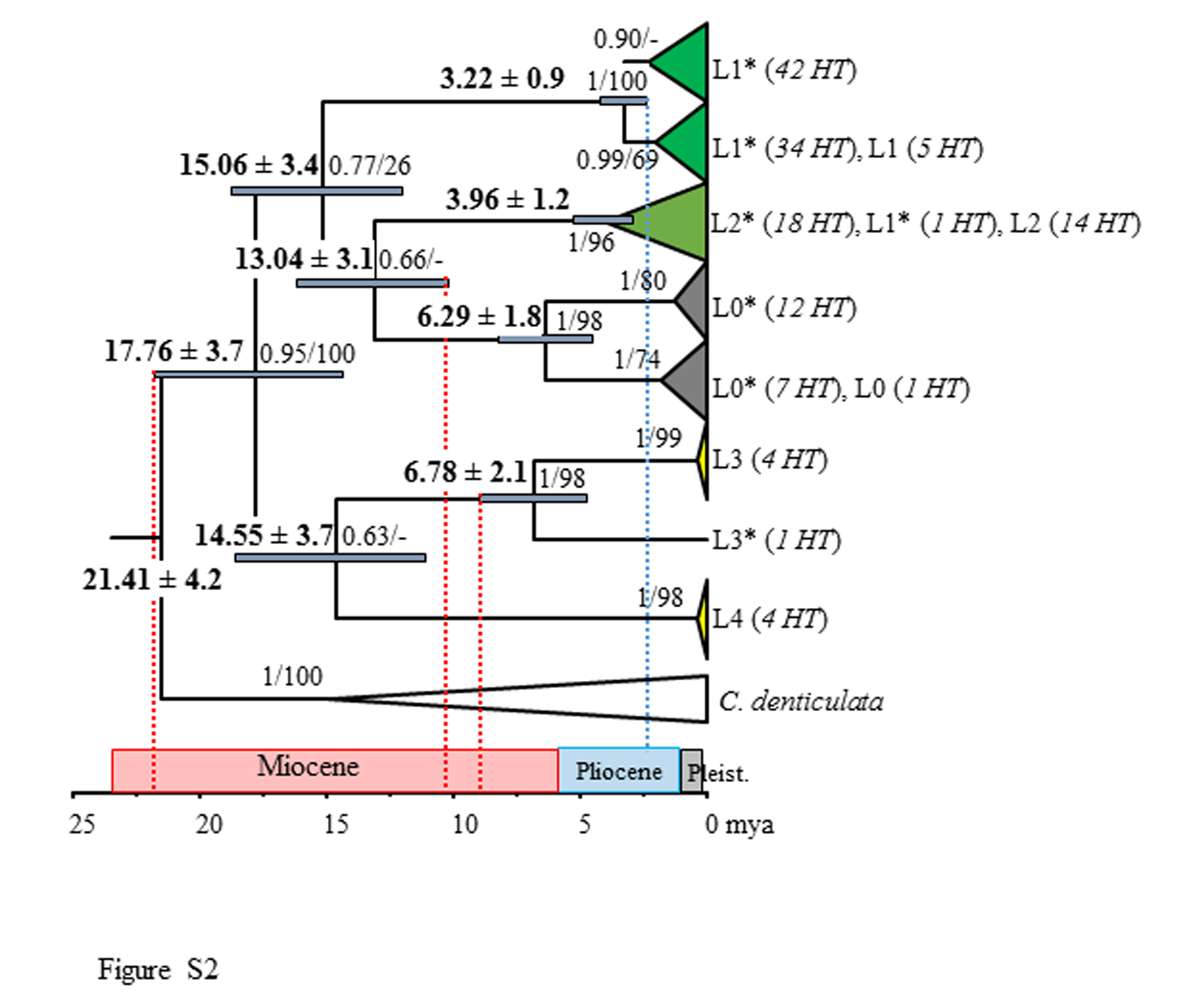

Supplement: S2 Fig — Molecular divergence estimates of European of P. notabilis calculated with BEAST based on a 500 bp alignment including all COI sequences from this study (n = 120) and from Porco et al. (2012) (n = 123). Numbers of different haplotypes (HT) are indicated in brackets next to the genetic lineage, * indicate lineages and haplotypes from Porco et al. (2012); dashed lines indicate radiations of major lineages in the Miocene (red) and Miocene-Pliocene (blue). Note that divergence estimates are several million years older than in 2B. (TIF) [file pone.0170909.s002.tif]

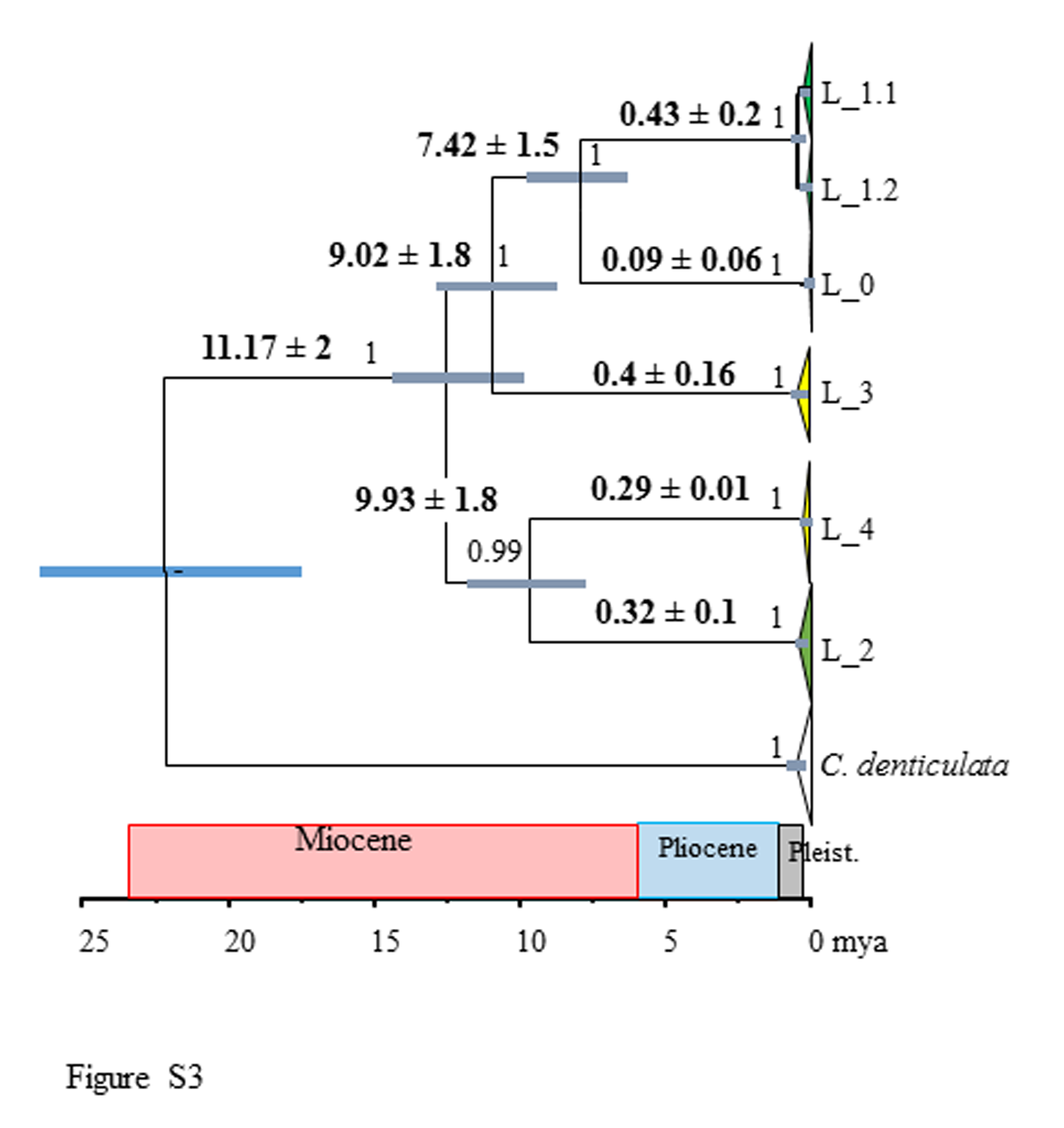

Supplement: S3 Fig — The combined alignment included 28S rDNA, COI and H3, for age estimation we useda strict clock with a substitution rate of 2.3% for COI and estimated substitution rates for the other genes. The topology differs slightly from the COI based phylogenetic trees but divergence estimates are very similar to those calculated with the 709 bp COI fragment presented in. 2B. (TIF) [file pone.0170909.s003.tif]

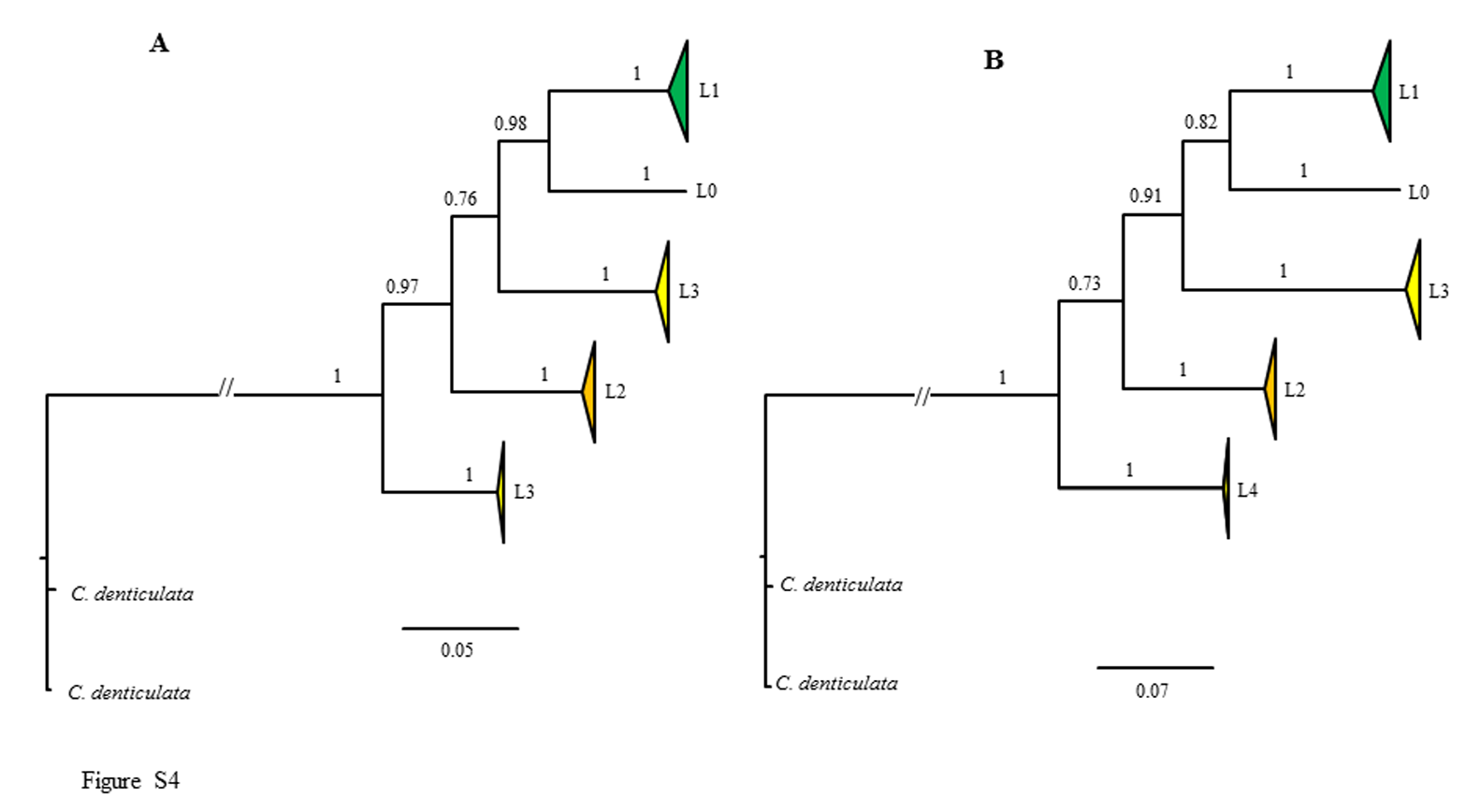

Supplement: S4 Fig — (A) The M3 model applied to COI and H3, the mitochondrial code was set for the COI partition and the universal code for H3 (mrbayes block settings: outgroup 43; charset D3 = 1–577; charset COI = 578–1285; charset H3 = 1286-.; partition by_gene = 3: D3, COI, H3; set partition = by_gene; lset applyto = (1) nucmodel = 4by4 code = universal nst = 6 rates = gamma; lset applyto = (2) code = metmt rates = invgamma; lset applyto = (3) code = universal nst = 1; lset applyto = (2,3) nucmodel = codon omegavar = M3; mcmc ngen = 1000000 samplefreq = 100; end; (B) The codon model M3 applied only for the nuclear coding gene (H3), for the mitochondrial COI gene the third codon position was excluded; this speeded up calculation times considerably, compared to the model of A (mrbayes block settings: exclude 578–1285\3; lset applyto = (1, 3) code = universal; lset applyto = (1) rates = gamma; lset applyto = (1,2) nucmodel = 4by4 nst = 6; lset applyto = (2) code = metmt rates = invgamma; lset applyto = (3) nst = 1 nucmodel = codon omegavar = M3; mcmc ngen = 1000000 samplefreq = 100; end;). Settings of the Bayesian tree in Fig 2A were: lset applyto = (1) nst = 6 rates = gamma; lset applyto = (2) nst = 6 rates = invgamma; lset applyto = (3) nst = 1; mcmc ngen = 10000000 samplefreq = 1000; end; (TIF) [file pone.0170909.s004.tif]
