# Supplementary material for: Genetic structure and distribution of Parisotoma notabilis (Collembola) in Europe: Cryptic diversity, split of lineages and colonization patterns
Source: PLoS One. 2017 Feb 7;12(2):e0170909. doi: 10.1371/journal.pone.0170909 (PMC5295681; doi:10.1371/journal.pone.0170909)
Supplement: S1 Table — All sequences are available at NCBI GenBan. Countries, sampling locations and sampling coordinates are listed. (PDF) [file pone.0170909.s005.pdf]

S1 Table. Accessionnumbers of DNA sequences of *Parisotoma notabilis* from Europe obtained in this study. All sequences are available at NCBI GenBank.Countries, sampling locations and sampling coordinates are listed.

country	location	coordinates (N, E)	abbre-viation	*COI*	*H3*	28S
**Bulgaria**	Bosnek	42.50° 23.17°	BG	KJ792225-9	KJ792345-9	KJ792105-9
**Gemany**	Uelzen	52.93° 10.61°	DE1	KJ792263-7	KJ792383-7	KJ792143-7
	Norden	53.58° 7.24°	DE2	KJ792268-71	KJ792388-91	KJ792148-51
	Solling, Neuhaus	51.71° 9.64°	DE3	KJ792272-6	KJ792392-6	KJ792152-6
**Denmark**	Humlebaek	52.93° 10.61°	DK	KJ792235-9	KJ792355-9	KJ792115-9
**Spain**	Oviedo	43.36° -6.00°	ES1	KJ792324-6	KJ792444-6	KJ792204-6
	Ponga	43.19° -5.16	ES2	KJ792327-31	KJ79247-51	KJ792207-11
**France**	Voegtlinshoffen	48.02° 7.27°	FR1	KJ792240-3	KJ792360-3	KJ792120-3
	Salavas	44.39° 4.37	FR2	KJ792249-53	KJ792369-73	KJ792129-33
	Chartreuse	45.42° 5.81°	FR3	KJ792254-8	KJ792374-8	KJ792134-8
	Rambouillet	48.62° 1.86°	FR4	KJ792259-62	KJ792379-82	KJ792139-42
	Korsika, Olmi-Capella	42.52° 9.02°	FR5	KJ792244-8	KJ792364-8	KJ792124-8
**Great Britain**	Melrose	53.58° 7.24°	GB1	KJ792309-13	KJ792429-33	KJ792189-93
	Island of Arran	51.71° 9.64°	GB2	KJ792314-8	KJ792434-8	KJ792194-8
**Greenland**	Kobbefjord / Nuuk	64.16° -51.52°	GL	KJ792277-81	KJ792397-401	KJ792157-61
**Greece**	Chrysovitsi	37.56° 22.20°	GR	KJ792282-6	KJ792402-6	KJ792162-6
**Croatia**	Sljeme	45.90° 15.95°	HR	KJ792230-4	KJ792350-4	KJ792110-4
**Italy**	Felitto	40.37° 15.22°	IT	KJ792287-90	KJ792407-10	KJ792167-70
**Norway**	Rod	59.07° 10.23°	NO1	KJ792291-4	KJ792411-4	KJ792171-4
	Skjervenmoen, Fössa Öst	59.45° 10.07°	NO2	KJ792295-8	KJ792415-8	KJ792175-8
**Serbia**	Sreckovac	43.03° 22.69°	RS	KJ792319-23	KJ792439-43	KJ792199-203
**Russia**	Petrozavodsk, Karelia	61.77° 34.22°	RU1	KJ792299-303	KJ792419-23	KJ792179-83
	Znamenskoe	55.73° 37.18°	RU2	KJ792304-8	KJ792424-8	KJ792184-8
**Slovenia**	Postojna	45.76° 14.21°	SI	KJ792332-4	KJ792452-4	KJ792212-4
**Turkey**	Kayseri	38.67° 35.53°	TR	KJ792335-9	KJ792455-9	KJ792215-9
**Ukraine**	Kubalach, Crimea	45.00° 34.82°	UA	KJ792340-4	KJ792460-4	KJ792220-4
