# Supplementary material for: Genetic structure and distribution of Parisotoma notabilis (Collembola) in Europe: Cryptic diversity, split of lineages and colonization patterns
Source: PLoS One. 2017 Feb 7;12(2):e0170909. doi: 10.1371/journal.pone.0170909 (PMC5295681; doi:10.1371/journal.pone.0170909)
Supplement: S2 Table — Lineages correspond to nodes connecting clades in Fig 2B, Lineages 1.1 and 1.2 are the two sister clades of Lineage 1 with 26 (L1.1) and 19 (L1.2) individuals. Nucleotide diversity (π), pairwise difference between sequences (d), number of individuals (n), sequence length (L) and nucleotide diversity estimator (θ). Calculations are based on genetic distances and the phylogeny generated with 120 individuals and a 709 bp fragment of COI. The estimator of nucleotide diversity θ was calculated as π/(1–4π/3). (PDF) [file pone.0170909.s006.pdf]

S2 Table. Values to estimate speciation among parthenogenetic lineages (Birky's 4x rule) for the five genetic lineages of *Parisotoma notabilis* sampled in Europe. Lineages correspond to nodes connecting clades in Figure 2B, Lineages 1.1 and 1.2 are the two sister clades of Lineage 1 with 26 (L1.1) and 19 (L1.2) individuals. Nucleotide diversity (π), pairwise difference between sequences (d), number of individuals (n), sequence length (L) and nucleotide diversity estimator (θ). Calculations are based on genetic distances and the phylogeny generated with 120 individuals and a 709 bp fragment of COI. The estimator of nucleotide diversity θ was calculated as π/(1-4π/3).

	**π**	**θ**	**d**	**n**	**L**
**Lineage 0**	0.00	0.00	0.00	12	709
**Lineage 1.1**	2.35	-0.84	2.26	26	709
**Lineage 1.2**	0.37	-2.32	0.35	19	709
**Lineage 2**	3.51	-0.81	3.44	53	709
**Lineage 3**	3.00	-0.82	2.40	5	709
**Lineage 4**	2.5	-0.83	2.00	5	709
**Lineage1**	4.78	-0.75	4.67	45	709
**Lineage 0-1**	41.52	-0.75	40.79	57	709
**Lineage 0-2**	69.75	-0.75	69.12	110	709
**Lineage 3-4**	71.58	-0.75	64.42	10	709
