# Supplementary material for: Genetic structure and distribution of Parisotoma notabilis (Collembola) in Europe: Cryptic diversity, split of lineages and colonization patterns
Source: PLoS One. 2017 Feb 7;12(2):e0170909. doi: 10.1371/journal.pone.0170909 (PMC5295681; doi:10.1371/journal.pone.0170909)
Supplement: S3 Table — K/θ ≥ 4 indicate that samples are from different species and K/θ ≤ 4 indicate that samples are from the same species. K is the observed sequence distance d from S2 Table corrected for multiple hits using GTR+G+I as estimated by TOPALI.θ values correspond to S2 Table, if θ of two clades differed, the larger value was used. (PDF) [file pone.0170909.s007.pdf]

S3 Table. K/θ between highly supported clades of *Parisotoma notabilis* from Europe to estimate K for Birky's 4x rule. K/θ ≥ 4 indicate that samples are from different species and K/θ ≤ 4 indicate that samples are from the same species. K is the observed sequence distance d from S2 Table corrected for multiple hits using GTR+G+I as estimated by TOPALI.θ values correspond to S2 Table, if θ of two clades differed, the larger value was used.

	**K/θ**	**K**
**L1.1/L1.2**	-3.34	7.75
**L1.1/L0**	-135.26	113.62
**L1.2/L0**	-47.32	109.79
**L1/L0**	-149.33	112
**L0-L1/L2**	-140.80	114.05
**L3/L4**	-152.27	114.2
**L0-2/L3-4**	-160.88	120.66
