# Supplementary material for: Genetic structure and distribution of Parisotoma notabilis (Collembola) in Europe: Cryptic diversity, split of lineages and colonization patterns
Source: PLoS One. 2017 Feb 7;12(2):e0170909. doi: 10.1371/journal.pone.0170909 (PMC5295681; doi:10.1371/journal.pone.0170909)
Supplement: S4 Table — For COI, the amino acid alignment (aa) was analyzed, for Histone 3 the nucleotide (nct) alignment was investigated as all amino acid sequences were identical. The common characters (amino acid for COI, nucleotide for Histone 3 and 28S rDNA) are listed next to the specific substitution. (PDF) [file pone.0170909.s008.pdf]

S4 Table. Positions of lineage specific substitutions in the alignments of three genes (*COI, Histone 3*, 28S rDNA) of 120 individuals of *Parisotoma notabilis* sampled across Europe. For *COI*, the amino acid alignment (aa) was analyzed, for *Histone 3* the nucleotide (nct) alignment was investigated as all amino acid sequences were identical. The common characters (amino acid for *COI*, nucleotide for *Histone 3* and 28S rDNA) are listed next to the specific substitution.

COI	**position**	**lineage**	**specific aa**	**common aa**	**specific haplotypes***
	360-362	L0	I	V	
	147-149	L1	A	M	2-4, 6
		L1	T	M	8-9
	498-500	L1	A	V	
	81-83	L2	A	V	
		L2	T	V	16-17, 22
	507-509	L3	K	T	
	48-50	L4	I	V	
Histone H3	**position**	**lineage**	**specific aa**	**common aa**	
	52-54	L4	R	S	
	127-129	L4	R	S	
Histone H3	**position**	**lineage**	**specific nct**	**common nct**	
	117	L0	G	C	
	228	L0	G	C	
	234	L0	T	C	
	351	L0	G	A	
	138	L1	C	T	
	159	L1	G	T	
	117	L2	G	C	
	243	L2	T	G	
	270	L2	C	G	
	279	L2	C	T	
	309	L2	A	T	
**28S rDNA D3-D5**	**position**	**lineage**	**specific nct**	**common nct**	
	122	L0	C	T	
	124	L0	C	T	
	133	L0	A	T	
	144	L0	T	A	
	122	L1	C	T	
	132	L1	T	C	
	135	L1	C	T	
	142	L1	C	A	
	147	L1	C	T	
	158	L2	A	G	
	116	L2	A	T	
	119	L2	G	A	
	119	L4	G	A	
	124	L4	T	C	
	125	L4	T	gap	
	130	L4	A	G	
	148	L4	T	C	
	154	L4	A	G	

*sampling locations of specific haplotypes (no. of individuals), see also Table S1

L1_2-4, 6	BG (3), DE1 (4), DK (2), FR2 (4), IT (1), RS (1), RU2 (5), TR (1), UA (1)
L1_8-9	TR (4)
L2_16-17, 22	FR1 (4), FR4 (4)
